# Validation of the multimedia version of the RDC/ TMD axis II questionnaire in Portuguese

**DOI:** 10.1590/S1678-77572010000300006

**Published:** 2010

**Authors:** Ricardo Figueiredo CAVALCANTI, Luciana Moraes STUDART, Maurício KOSMINSKY, Paulo Sávio Angeiras de GOES

**Affiliations:** 1 DDS, Dental School, University of Pernambuco, Camaragibe, PE, Brazil.; 2 DDS, Graduate student in Science of the Language, Catholic University of Pernambuco, Camaragibe, PE, Brazil.; 3 PhD, Senior Lecturer, Discipline of Restorative Dentistry, Dental School, University of Pernambuco, Camaragibe, PE, Brazil.; 4 PhD, Senior Lecturer, Discipline of Public Health, Dental School, University of Pernambuco, Camaragibe, PE, Brazil.

**Keywords:** Computer-assisted, diagnosis, Temporomandibular joint dysfunction syndrome, Validation studies

## Abstract

**Objective:**

The aim of the study was to validate the multimedia version of the Research Diagnostic Criteria for Temporomandibular Disorders (RDC/TMD) Axis II Questionnaire in Portuguese language.

**Material and methods:**

The sample comprised 30 patients with signs and symptoms of temporomandibular disorders (TMD), evaluated at the Orofacial Pain Control Center of the Dental School of the University of Pernambuco, Brazil, between April and June 2006. Data collection was performed using the following instruments: Simplified Anamnestic Index (SAI) and RDC/TMD Axis II written version and multimedia version. The validation process consisted of analyzing the internal consistency of the scales. Concurrent and convergent validity were evaluated by the Spearman’s rank correlation. In addition, test and analysis of reproducibility by the Kappa weighted statistical test and Spearman´s rank correlation test were performed.

**Results:**

The multimedia version of the RDC/TMD Axis II questionnaire in Portuguese was considered consistent (Crombrach alpha = 0.94), reproducible (Spearman 0.670 to 0.913, p<0.01) and valid (p<0.01).

**Conclusion:**

The questionnaire showed valid and reproducible results, and represents an instrument of practical application in epidemiological studies of TMD in the Brazilian population

## INTRODUCTION

Temporomandibular disorders (TMD) is a collective term embracing a number of clinical problems that involve the masticatory musculature, the temporomandibular joint or both^11^. Most diagnosed patients suffer from muscle and/or joint pain on palpation and/or mandibular movements^13^.

Epidemiological studies on TMD have shown conflicting results regarding prevalence and incidence. Such findings could be attributed to different diagnostic criteria. Thus, a standardized methodology is necessary when comparing studies related to these diseases^6, 10^.

The contradictory findings may be attributed in part to the lack of standardized diagnostic criteria for defining clinical subtypes of TMD and for psychological assessment. In order to overcome this difficulty, Dworkin and LeResche^2^ (1992) developed a set of diagnostic tools for TMD, namely the Research Diagnostic Criteria for Temporomandibular Disorders (RDC/TMD), including clinical aspects (Axis I) and psychological/psychosocial factors (Axis II). Axis I permits the reproducibility of the diagnostic criteria for research on TMD in their most common forms of muscle and joint involvement, while Axis II enables the physical diagnosis to be coordinated with an operational assessment of the psychological distress and psychological dysfunction associated with TMD^2, 3, 12, 17^.

The production of the RDC/TMD represented an advance in the study of this subject^2,16^. Although RDC/TMD is a diagnostic system that evaluates only the most common forms of TMD^14,16^ , it provides specifications for conducting a standardized clinical physical examination. It has been formally translated/back-translated into 20 languages and is the most common diagnostic method used by the 45-member consortium of RDC/TMD-based international researchers (International Consortium for RDC/TMD-based Research, 2004). Recent studies have demonstrated the validity and reliability of the clinical diagnosis of TMD^8,13^. Furthermore, several studies of the validation of diagnosis in TMD have focused on RDC, comparing its consistency with that of other imaging techniques^13^.

Due to the importance of psychosocial factors in the diagnosis of TMD, the time lag between the examination of the patient and the collection of data has proved to be a problem. With a view to solving this problem, Yap, et al.^18^ (2001) developed an on-line computerized version of the RDC/ TMD questionnaire that, despite facilitating the diagnosis, encountered a number of operational problems involving the patient’s difficulties in answering the questions and using the computer.

After the initial process, a final procedure of validation was carried out, as a result the RDC/ TMD PORTUGUESE VERSION became available to be used in studies conducted in Brazilian population.

The aim of this prospective study was to validate the Brazilian Portuguese-language multimedia version of the RDC/TMD Axis II Questionnaire, a standardized form of research on TMD, enhancing the interaction of clinicians and researchers.

## MATERIAL AND METHODS

The present multimedia version of the Questionnaire Axis II of the RDC/TMD followed the written version of the questionnaire in Portuguese validated by Lucena, et al.^9^ (2006). It runs on Windows OS 2000 and XP and can exploit multimedia software and data storage bank capabilities.

The questionnaire was designed in such a way that the patient could hear the video presentation and visualize the question, so that at the end of each presentation, the respondent could answer the question, replay it, or go back. The types of questions included yes/no questions and multiple-choice questions. The Graduated Chronic Pain Scale (GCPS) was used for the questions measuring the intensity of pain.

The study population consisted of patients with orofacial pain seeking treatment at the Orofacial Pain Control Center of the Dental School of the University of Pernambuco, Brazil, between April and June 2006. The ages of the subjects ranged from 19 to 55 years (mean = 39.5 years), 93.3% of whom were females and 6.7% males. All patients had TMD. The inclusion criteria were no previous history of TMD treatment and a positive diagnosis of TMD, using the Simplified Anamnestic Index (SAI)^5^. The sociodemographic data of all the subjects with TMD are presented in Table 1.

**Table 1:** Sociodemographic data and pain-related statistics of TMD patients

**DEMOGRAPHIC VARIABLE**	**n**	**%**
		
**Gender**		
Female	28	89.0
Male	2	11.0
**Age (yrs)**		
Mean	39.5	-
Standard Deviation	11.6	-
**Marital status**		
		
Married	16	53.3
Single	7	23.3
Cohabiting	4	13.3
Separated	3	10.0
		
Patients with complete secondary education	15	50.0
		
**RDC/TMD diagnosis:**		
Pain intensity and disability		
Group I	2	6.7
Group II	10	33.3
Group III	0	0
Group IV	18	60.0
**Depression**		
Normal	8	26.7
Moderate	13	43.3
Severe	5	16.7

The research project involving research on humans was submitted to the Research ethics Committee of the University of Pernambuco after granting the permission from the Dental School. Only patients who agreed to participate and signed the informed consent form were enrolled in the study.

The following questionnaires were answered by the participants: 1) SAI^5^ questionnaire, which verified the presence of signs and symptoms of TMD, enabling a prompt diagnosis. All patients were classified as having TMD. 2) Portuguese-language written version of the RDC/TMD Axis II Questionnaire^10^ , which consisted of 31 items, divided into sociodemographic, socioeconomic, psychological and psychosocial factors, signs and symptoms, and scale of limitation of mandibular function. 3) Portuguese-language multimedia version of the RDC/TMD Axis II Questionnaire.

The questionnaires were applied to the 30 participants one day after the initial interview as follows: 15 were given the written questionnaire and 15 the multimedia questionnaire for the purpose of testing the validity by means of process test/retest the instrument. None of the patients had received any previous treatment for any associated comorbidity, any pharmacological or psychological therapy for TMD. All of them answered the SAI questionnaire.

The validation process consisted of verifying the internal consistency of the scales of limitation of mandibular function and psychological factors. In order, to have an acceptable internal consistency, a scale should present alpha values of at least 0.7. Convergent validity was simultaneously evaluated by Spearman’s rank correlation test^10^. The reproducibility analysis used the weighted Kappa statistical test and Spearman’s rank correlation test (µ>0.05)^13^. The Statistical Package for the Social Sciences (SPSS) for Windows, Version 10, was used for descriptive and inferential analysis of the data. The level of statistical significance was set at 5%. Internal consistency was estimated by Cronbach’s alpha test, values above 0.5 being considered satisfactory^14^.

## RESULTS

The time taken by the patients who answered the multimedia questionnaire ranged from 15 to 40 min (mean time of 25 min). By comparing the information obtained from 30 patients submitted to evaluations by two exams with the two versions of the RDC/TMD questionnaire at different times (study test-retest), a consistent and reproducible instrument was identified for obtaining a diagnosis.

The analysis of the internal consistency of the limitation of mandibular function as a RDC/TMD Axis II diagnostic criterion identified 12 items that correlated with each other and with the overall result of the scale, comprising a true scale, with alpha=0.71 and standardized alpha=0.70 (Table 2).

**Table 2:** Internal consistency of limitations related to mandibular functioning values of RDC/TMD Axis II, evaluated by test-retest in 30 patients

**LIMITATION RELATED TO MANDIBULAR FUNCTIONING RDC/TMD AXIS II**	**STATISTICAL PARAMETERS IF ITEM DELETED**		
	**Scale**	**Corrected**	**Alpha if Item**
	**Variance**	**Item-Total **	**Deleted**
		** Correlation**	
			
Chewing	5.9	0.28	0.7
Drinking	4.86	0.61	0.64
Exercising	5.2	0.43	0.68
Eating hard foods	6.1	0.14	0.71
Eating soft foods	5.44	0.42	0.68
Smiling/laughing	5.63	0.35	0.69
Sexual activity	5.96	0.13	0.72
Cleaning teeth or face	4.99	0.53	0.66
Yawning	5.97	0.19	0.71
Swallowing	5.15	0.45	0.67
Talking	5.2	0.43	0.68
Having your usual facial appearance	4.99	0.53	0.66

NOTE: alpha (α) = 0.71 standardized item alpha (α) = 0.70

In addition to the internal consistency of the questions related to psychological factors, comprising 32 items, which also correlated with one another, since the alpha values exceeded 0.90 (alpha=0.94 and standardized alpha=0.94) (Table 3). Therefore, the alpha values for scale were not increased by the exclusion of any of the items tested.

**Table 3:** Internal consistency of the scale of psychological factors values of RDC/TMD Axis II, evaluated by test-retest in 30 patients

	**STATISTICAL PARAMETERS IF ITEM DELETED**		
**PSYCHOLOGICAL FACTORS RDC/TMD AXIS II**			
	**Variance**	**Corrected**	**Alpha if Item**
		**Item-Total **	**Deleted**
		** Correlation**	
			
Headaches	572.3034	0.4383	0.9415
Loss of sexual interest	563.6276	0.4343	0.9418
Faintness/dizziness	569.6885	0.5012	0.9410
Pains in the heart/chest	560.8609	0.5521	0.9405
Feeling low in energy	549.9092	0.7662	0.9385
Thoughts of dying	552.4609	0.6062	0.9400
Poor appetite	562.5989	0.4672	0.9414
Crying easily	551.4264	0.7208	0.9389
Blaming yourself for things	557.1138	0.6077	0.9400
Pains in the lower back	579.2920	0.2294	0.9436
Feeling lonely	557.7885	0.5535	0.9405
Feeling blue	557.0678	0.7068	0.9392
Worrying too much about things	569.3161	0.5636	0.9406
Feeling no interest in things	560.7828	0.5011	0.9410
Nausea or upset stomach	582.7138	0.1726	0.9442
Soreness of your muscles	559.5586	0.6221	0.9399
Trouble falling asleep	549.7345	0.6501	0.9395
Trouble getting your breath	557.7057	0.5270	0.9408
Hot or cold spells	563.0586	0.5207	0.9408
Numbness in parts of your body	561.7069	0.5444	0.9406
A lump in your throat	557.4816	0.5913	0.9401
Feeling hopeless about future	554.9023	0.6440	0.9396
Feeling weak in parts of your body	553.0126	0.6473	0.9395
Heavy feeling in your arms or legs	555.6276	0.7158	0.9391
Thoughts of ending your life	568.5103	0.4836	0.9411
Overeating	552.0230	0.5991	0.9400
Awakening in the early morning	547.6506	0.7300	0.9387
Sleep that is restless or disturbed	545.6828	0.7951	0.9381
Feeling everything is an effort	562.4609	0.5544	0.9405
Feelings of worthlessness	550.4644	0.6206	0.9398
Feeling of being caught or trapped	565.0678	0.4864	0.9411
Feelings of guilt	554.2862	0.5977	0.9400

NOTE: alpha (α) = 0.942 standardized item alpha (α) = 0.943

The multimedia RDC/TMD Questionnaire proved to be reproducible, presenting correlations greater than µ>0.05, all of which showed statistical significance when evaluated at different times (Spearman 0.670 to 0.745, p<0.01) (Table 4).

**Table 4:** Reproducibility of the questions and of established diagnostic procedures in RDC/TMD Axis II, evaluated by test-retest of 30 patients

**REPRODUCIBILITY FROM RDC/TMD **	**STATISTICAL TESTS **	**INTERPRETATION **
		
**Axis II - Questions**	**Spearman's Correlation (μ)**	**p value**
		
Degree of satisfaction with one's general health	0. 670	< 0.01
Degree of satisfaction with one's oral health	0.741	< 0.01
Orofacial pain at the time of the study	0.708	< 0.01
Worst orofacial pain in the last six months	0.745	< 0.01
**Diagnosis from RDC/TMD**		
Pain intensity and disability	0.883	< 0.01
Depression	0.913	< 0.01
Nonspecific physical symptoms		
pain items included	0.715	< 0.01
pain items excluded	0.706	< 0.01

The reproducibility of the different domains of RDC/TMD Axis II, considering the severity of chronic pain and the three subscales of psychological factors (depression, nonspecific physical symptoms with and without pain items), was analyzed. Considering the information obtained from the 30 patients (testretest study), there was a statistically significant diagnostic agreement in Axis II (µ ≥0.7; p<0.01) (Table 4).

## DISCUSSION

Patients younger than 18 years were excluded from the study because many questions are difficult for them to understand or inappropriate and because the RDC/TMD has been calibrated with data only on patients over the age of 18 years^18^.

The size of the study sample was based on validation and cultural adaptation studies, using groups with 10 to 37 patients^1,9,15^.

The Portuguese-language written version of the RDC/TMD Axis II Questionnaire was validated by Lucena, et al.^9^ (2006) using criteria established in similar studies^1,3,7^ , generating a reliable and reproducible instrument, as estimated by the Kappa statistical test and consistent on Cronbach’s alpha reliability test.

The mean age of the patients in this study was 39.5 years, which corresponds to that found in most studies on TMD^3,4,8-10,16-19^. With regard to gender, in the studied population, 28 (93.3%) were females and 2 (6.7%) males. Despite the fact that several studies report a predominance of females with TMD, this predominance in our group was above average^3,4,8-10,16-19^.

In this study the assessment of the internal consistency of the scales of limitation of mandibular function and psychological factors presented an overall alpha index of 0.71 and 0.94, respectively. The lowest value used as baseline in clinical studies is 0.7^13^. Thus, the internal consistency of the scales was valid, resulting in a true scale. Spearman’s ranks test demonstrated that the proposed version of the instrument generally had a positive correlation, indicating that the validated version is indeed measuring what it was intended to measure^14^.

This study represents an important step for research on TMD in Brazil in several aspects. Firstly, it represents an advance in relation to the method of research previously reported^15^ in the study of the TMD; in addition, it will facilitate future population studies involving TMD. As a result, the multimedia version will reduce the cost of printing, making copies of the questionnaire and storing patient data.

The present multimedia questionnaire also permits greater speed in the collection of patient data, storage and statistical analysis, with information being simultaneously entered in a database while the research is being conducted. It also has the advantage of eliminating any bias on the part of the interviewer when dealing with sensitive questions. However, results must be interpreted considering its limitations, as it has been carried out in a specialized pain clinic and with a limited sample of patients. Future investigation in a broad epidemiological survey should confirm the results.

## CONCLUSION

The process of validating the Portuguese multimedia version of the RDC/TMD Axis II Questionnaire followed the methodology proposed in the literature and resulted in a reproducible and readily applicable instrument, valid for the Brazilian population, making it possible to introduce an innovative standardized method in epidemiological studies on TMD in Brazil.
